# Comparison of clear cell carcinoma and benign endometriosis in episiotomy scar - two cases report and literature review

**DOI:** 10.1186/s12905-020-0880-5

**Published:** 2020-01-20

**Authors:** Song Xu, Wei Wang, Li Ping Sun

**Affiliations:** 1 Department of Gynecology, Affiliated Hangzhou First People’s Hospital, Zhejiang University School of Medicine, NO.261 HuanSha Road, ShangCheng District, Hang Zhou, 310006 Zhe Jiang Province China; 2 Department of Pathology, Affiliated Hangzhou First People’s Hospital, Zhejiang University School of Medicine, Hangzhou, 310006 China

**Keywords:** Endometriosis, Clear cell carcinoma, Episiotomy scar, Malignant transformation

## Abstract

**Background:**

Malignant endometriosis in an episiotomy scar is rare; only seven cases have been reported previously. Here, we compare two cases of benign endometriosis and clear cell carcinoma.

**Case presentation:**

The first case was a 54-year-old woman who presented with a large perineal lesion in her episiotomy scar with high 18F-fluorodeoxyglucose uptake. This location had a history of endometriosis many years ago. She underwent radical excision of the mass and bilateral inguinal lymph node dissection. Histological and immunohistochemical analysis confirmed the presence of clear cell carcinoma arising from endometriosis. Assisted radiotherapy was performed after surgery due to a positive lymph node. No recurrence was detected over a 1-year follow-up period. The second case deals with a 3 × 2 cm mass in the episiotomy scar of a 33-year-old woman. Part of the anal sphincter was resected because of the close proximity of the lesion. Because the disease lay very close to the anus, she received anal sphincter reconstruction combined with mass excision. Pathology result showed typical endometrial glands and interstitial tissues.

**Conclusions:**

Deleterious change only happens in patients experiencing perineal endometriosis. Complete excision is crucial for this form of disease; sometimes impairment of the anal sphincter is also necessary. Patients with malignancy required a combination of treatments in order to improve their prognosis.

## Background

Endometriosis is a common disease in women and is characterized by the presence of ectopic functional endometrial tissue as an inflammatory condition [1]. Endometriosis can occur at any location, but the incidence of perineal endometriosis is rare and is usually associated with previous episiotomy or other forms of vulvar surgeries [2]. Its mechanism may be the transplantation seeding of endometriosis [3]. Sometimes it is difficult to diagnose such cases without careful case history and clinical findings. The malignant transformation of endometriosis was first described by Sampson in 1925 [4], but cases of malignancy arising from scar endometriosis are extremely rare. The literature relating to this disease consists of only four cases of clear cell carcinoma and one patient with serous papillary cystadenocarcinoma [5–8]. Here, we report two cases of endometriosis with previous episiotomy scars, one was benign, the other was clear cell carcinoma. In this article, we summarize the clinical features and pathological findings for these patients.

## Case presentation

### Case 1

A 54-year-old female patient presented with a perineal lump which had gradually enlarged over a period of 4 years. Her obstetric history included a vaginal delivery with a left episiotomy 30 years ago. After that, she remained well for 3 years before she felt a small mass in the incision site. She then underwent resection of the mass and was diagnosed with endometriosis. However, this disease relapsed after a few months, and the patient suffered, and underwent the same operation again. After the second operation, she still felt incisional pain during menstruation. Danazol had been prescribed for 1 year, which relieved the pain. Following drug therapy, 18 years passed and the patient underwent radical mastectomy and chemotherapy due to breast carcinoma in 2012.

**Fig. 1:**
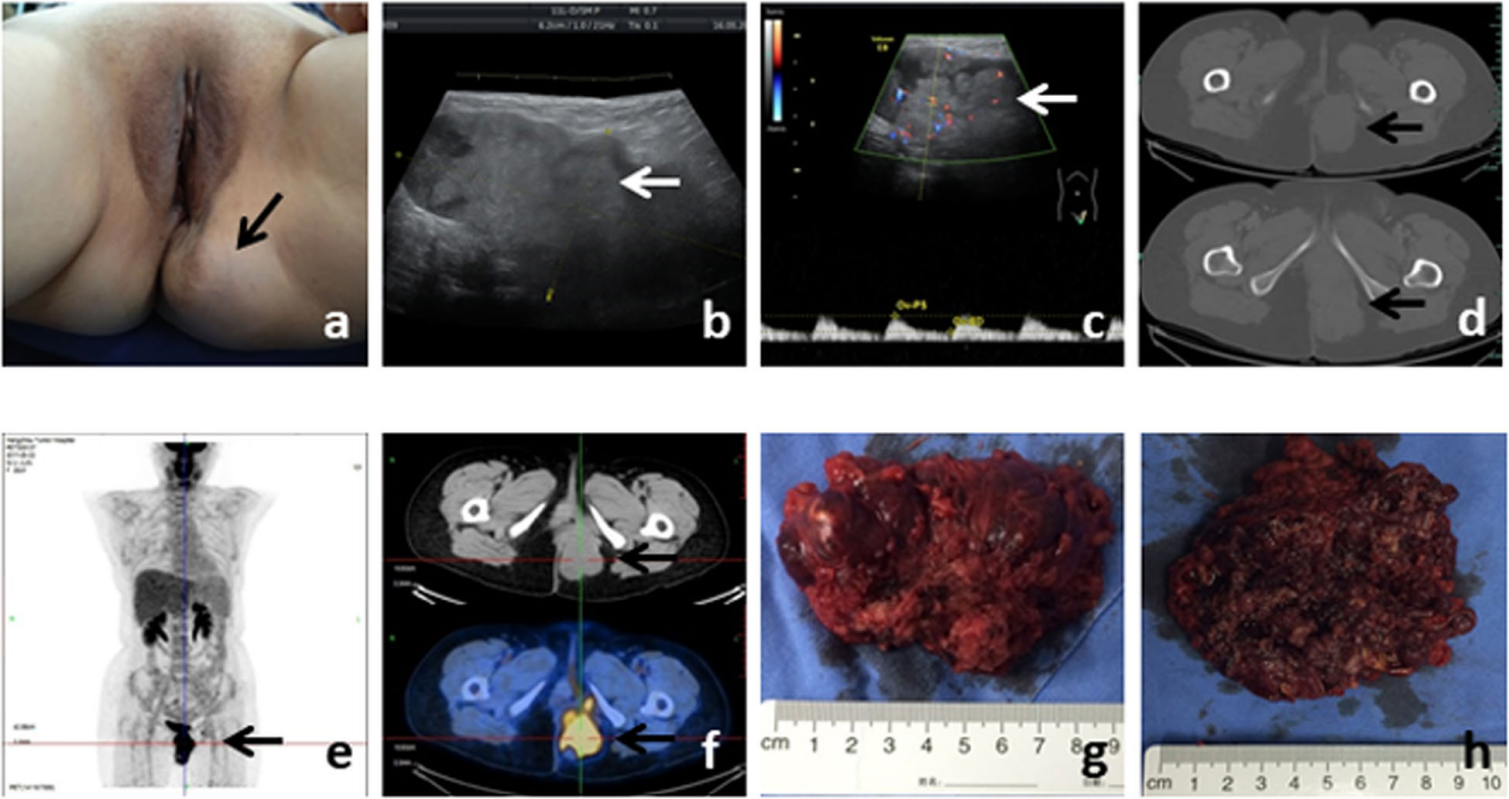
**a** Clinical appearance of the large mass. **b**, **c** Ultrasound and CDFI image of the perineal tumor, measuring 9.5 × 4.4 × 5.8 cm. **d** CT of recurrence at left perineum incision. **e**, **f** Appearance of the mass at PET/CT-scan, it showed an increased uptake of 18F-fluorodeoxyglucose. **g**, **h** Intact and dissected surgical specimen

Four years ago, she found a slow-growing perineal mass in the same region. She went to our hospital and physical examination revealed a soft mass, 6 × 5 cm in size, which was closely related to the anterior episiotomy scar (Fig. 1a). Color Doppler ultrasound revealed a well-defined mass in the left perineal area which measured 9.5 × 4.4 × 5.8 cm. Color Doppler flow imaging (CDFI) showed blood flow signals (Fig. 1b, c). Tumor markers CA125, AFP, CA-19-9, CEA, HE4 and SCCA were all normal. We performed enhanced pelvic computed tomography (CT) and identified an irregular soft tissue image between the left region of the anus and vagina (Fig. 1d). We also detected an enlarged lymph node in the left inguinal region. Both of these regions showed enhancement in the scan. Lymph node aspiration biopsy suggested epithelial neoplasm. Combined positron emission tomography and computed tomography (PET/CT) further showed focal increased 18F-fluorodeoxyglucose uptake in both of the tumor and the inguinal lymph node (Fig. 1e, f).

**Fig. 2:**
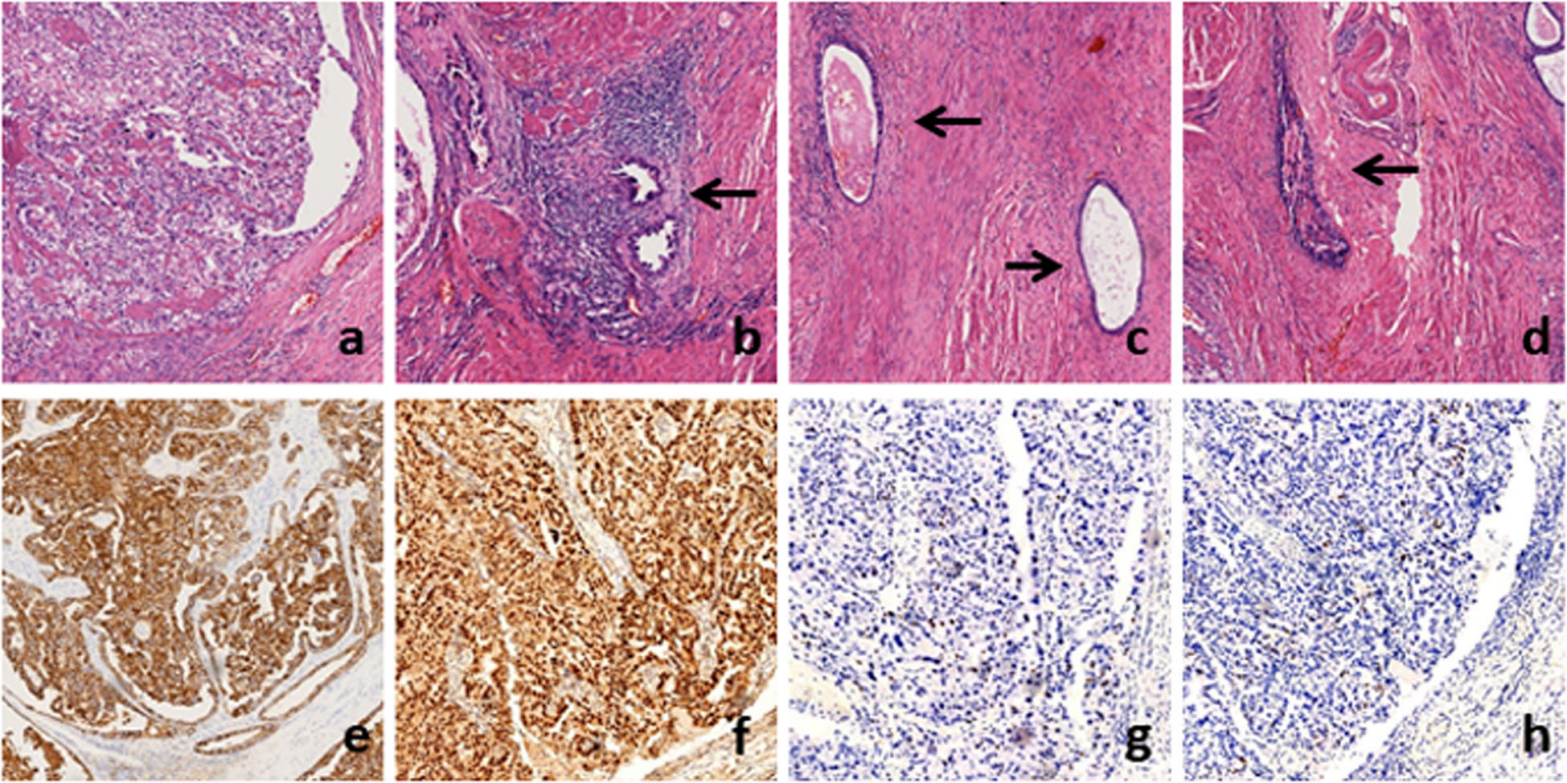
Microscopic images: **a** Clear cell carcinoma: marked papillary growth, abundance of cellulars and haemorrhage, necrosis, heteromorphism are manifest. **b** Typical hobnail cells could be found in our malignant case. **c** endometrial glands are noted in dense stroma. **d** The symbol of clear cell carcinoma arising from the endometriosis: Transitional zone. Confused structure of cells and disorder of nucleus. Immunohistochemical results demonstrated positve expression of CK and HNF1β (**e**, **f**), negenative expression of P53 and progesteron receptor (**g**, **h**)

We performed a radical excision of the tumor and bilateral inguinal lymph node dissection. During the surgery, we found the tumor was firm and tan or red-tan in color. The tumor had extended into the deep part of her pelvis, and the interface between it and the surrounding tissue was not well demarcated. Pathological analysis indicated clear cell carcinoma of the perineum incision, possibly arising from an endometrial implant (Fig. 2a-c). A transitional dysplastic zone between the endometriotic focus and the clear cell carcinoma was identified (Fig. 2d). Each side of the superficial inguinal region had one positive lymph node. Immunohistochemically, the tumor cells were positive for CK, P16, HNF1β and AMACR. The patient was discharged after 30 days of hospitalization without any complication. This was followed by 1 month of radiation therapy.

### Case 2

**Fig. 3:**
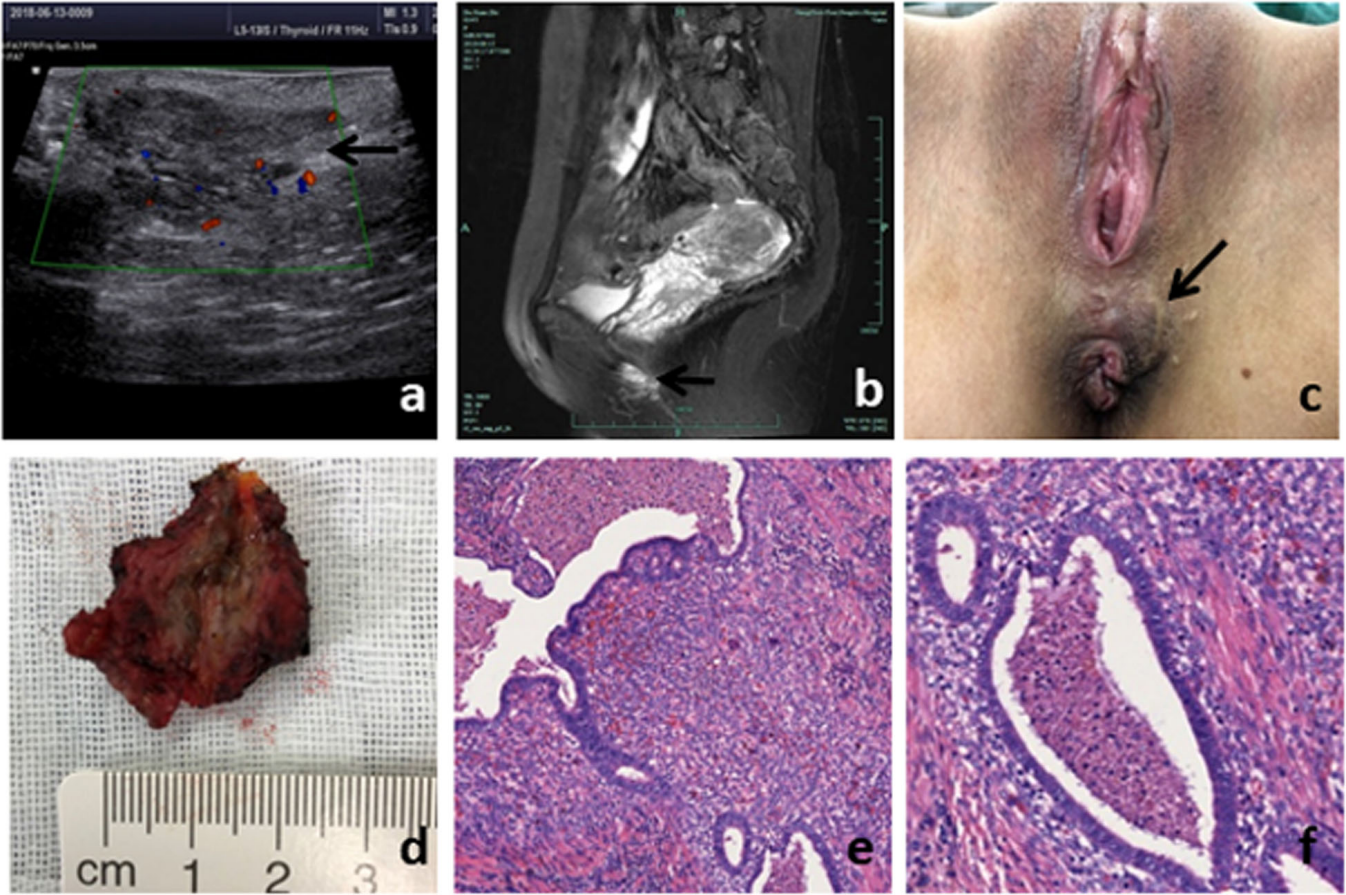
**a** Sonographic image showed a 3.1 × 1.6 cm hypoechoic lesion. **b** MRI image showed a subcutaneous solid mass near the left side of the anus on T2WI. **c** Dark nodule in left episiotomy scar. **d** Dissected surgical specimen showed a mass with old hemorrhage. **e**, **f** Endometrial glands with typical stroma were showed in histological examination

A 33-year-old Asian woman presented with a painful mass in her perineal incision, which she had noticed several months before. There was no particular medical history except that she had a vaginal delivery with episiotomy 12 years previously. Subsequently, she felt occasional incisional pain. Four years ago, she had another natural labor and the pain became worse. Over the last few months, the patient could feel a subcutaneous mass in the perineal incision. An ultrasound scan showed a hypoechoic nodule measuring 3.1 × 1.6 × 1.2 cm (Fig. 3a); its margin was clear and there was no enhanced spot echo. A minor blood flow signal was present on CDFI. Following the administration of intravenous contrast material, pelvic magnetic resonance imaging (MRI) revealed a sharply defined subcutaneous solid mass which was strictly adherent to the left side of the anus. (Fig. 3b). On palpation, a soft tumor was identified which lay in the midpoint of the scar (Fig. 3c). Serum levels of CA125, CEA and SCCA were normal, although CA199 was marginally increased at 41.5 Ku/l. At the end, the patient received a complete excision of the perineal mass that was carried out uneventfully. The focus appeared as a hard mass with an irregular shape and ill-defined margins. Part of the anal sphincter was resected because the lesion lay very close. The surgical specimen contained several brown capsulated tissues (Fig. 3d). Areas of necrosis, hemorrhage and cyst formation could be seen on sectioning. Histological examination revealed endometrial glands and a typical interstitium (Fig. 3e, f).

## Discussion and conclusions

Functioning endometrial tissue located outside of the uterine cavity is defined as endometriosis [9]. Cutaneous endometriosis is rare and mostly occurs in the abdominal wall, usually developing at the site of a caesarian scar. Perineal and vulvar lesions are more rare; the iatrogenic transplantation of endometrial cells via an episiotomy scar appears to be the mechanism involved [10]. Both of our cases support this hypothesis. These lesions appear as a blue-black nodule under the surgical scar, accompanied by cyclic pain and enlargement during menstruation. Three criteria for diagnosing malignant endometriosis were first proposed by Sampson in 1925: menstrual irregularity, a continuously enlarging mass and increased pain [4].

Tumor markers are not very sensitive for extraovarian lesions, even in malignant cases. Previously, Cuisenier reported that the levels of CA125 are normal in almost half of patients with extraovarian lesions but are normal in only 15.38% of cases with ovarian endometriosis [11]. Both of our patients had normal levels of CA 125. Ultrasound and MRI can help us to identify the exact anatomical position, and in our experience, MRI is highly sensitive and offers excellent differentiation of endometriosis from neighboring tissue; this is important for diagnosis and operative management. The differential diagnosis should include anal cancer, abscesses, fistula, atheroma and hidradenitis.

Malignant degeneration of cutaneous endometriosis is extremely rare, representing 0.3–1% of surgical scars [5]; and its origin is still unclear, although we know that some specific events occur in this process. On the one hand, the mutation of tumor suppressor genes, such as PTEN and P53 may be related to the development of endometriosis associated cancer [12]. The pathological results of our malignant patient were negative for P53, but positive for HNF1β and AMACR. On the other hand, hormones are also known to play a role. It has been confirmed that high levels of estrogen are consistent with the progression of endometrioid cancer and clear cell carcinoma. In addition, inflammatory reactions and cytokines such as IL-1, which can cause angiogenesis, proliferation and the inhibition of apoptosis, can also contribute to the development of this disease [13].

Benign and malignant perineal endometriosis are difficult to distinguish by symptoms or signs [14]. However, we identified a key point in that the malignant diseases are recurrent. The malignant patient in our report, and those in the existing literature, had undergone a perineal mass excision previously. This means that the initial case of this disease is always benign, but the transition of malignancy occurs after several years if the tumor relapses. Because all of the known malignant patients described in the literature, our present patient included, had undergone a resection previously, we suspect that the tumor had not been totally removed during the first surgery. After a long period of stimulation by local inflammation and hormones, these tumors ultimately became malignant. Thus, resection of the whole endometrial mass is crucially important during initial treatment, especially for patients with anal sphincter involvement. The diagnostic accuracy of anal sphincter involvement could be improved by the use of anal endosonography [3, 15]. Previous authors have recommended wide excision of endometrioma with primary sphincteroplasty for these patients [16], and that this is particularly important if the tumor boundary is unclear. Thus, a safe resection margin of more than 0.5 cm of surrounding healthy tissue is necessary in order to avoid relapse or malignancy. To achieve wide excision in cases of perineal endometrioma with anal sphincter invasion, partial removal of external anal sphincter is necessary [16, 17].

Some clinicians have suggested the use of oral drugs to manipulate hormone levels [18–20]. However, assisted drug therapy has not been proven to be effective in controlling or postponing the recurrence of perineal endometriosis [21]. For our patient, the use of danazol achieved symptomatic relief but did not prevent recurrence or malignant change. Because of the low incidence of perineal endometriosis, the ideal treatment is still unknown. Table 1 shows previously reported cases of malignant transformation of episiotomy scar endometriosis. In recent literature, a total of four patients with clear cell carcinoma and one patient with serous papillary cystadenocarcinoma have been reported [5–8, 22]. All of these patients had a history of benign perineal endometriosis. Methods of treatment include radical excision, radiotherapy and chemotherapy. Most of these patients received combined therapies. For our patient, we performed radical surgery and post-operative radiation. Although the results of long-term follow-up remain unknown, a 1 year period of follow up shows no recrudescence or metastasis.

**Table 1:** Summary of carcinoma cases derived from episiotomy scar

Author/Year	Age	Symptoms	Treatment	Histology	Follow-up
Hitti IF /1990 [9]	43	10 cm mass of right buttock	Biopsy, chemotherapy and radiotherapy	Clear cell carcinoma	Dead after 2.5 years
Todd RW/2000 [10]	54	3 cm mass in the rectovaginal pouch	Radiotherapy and chemotherapy	Clear cell carcinoma	Relapse after 6 months
Chene G/2007 [3]	50	perineal node of 3.5 cm in the right side of the anus	Radiotherapy, chemotherapy and complete excision	Serous papillary Cystadenocarcinoma	Relapse after 6 months
Kwon YS /2008 [11]	42	2.5 × 3.0 cm elevated cystic mass on the right mediolateral episiotomy	Radical excision	Clear cell carcinoma	No evidence of disease after 10 months
Han L /2016 [12]	36	10 × 5 cm mass in the apex of episiotomy	Radical excision and chemotherapy	Clear cell carcinoma	No evidence of disease after 6 months
Current report/2018	54	6 × 5 cm mass in episiotomy and 3 × 3 cm inguinal nodule	Radical resection and radiotherapy	Clear cell carcinoma	No evidence of disease after 15 months

Perineal endometriosis is rare but should be suspected if there is a history of episiotomy and cyclic pain. Ultrasonography, magnetic resonance imaging and PET/CT can be used for diagnosis. Complete resection of the nidus is key to treating this disease because malignancy only happens in recurrent patients. To achieve this, we should consider two key points: (1) adequate and wide excision is the principle of management to prevent recurrence and future malignancy; and (2) we must be very careful not to rupture tumors during surgery as this can cause remnants to remain and subsequent re-implantation to occur. The effect of assisted drug therapy after surgery is not very clear. Adjunctive chemotherapy and radiotherapy are recommended as the prognosis may be improved in malignant patients. Finally, it is very important to follow-up these patients with care.

## Data Availability

The datasets used and analysed during the current study are available from the corresponding author upon reasonable request.

## Abbreviations

AFP: Alpha fetoprotein
AMACR: Alpha-methylacyl-CoA racemase
CA125: Cancer antigen 125
CA199: Carbohydrate antigen 19–9
CEA: Carcinoembryonic antigen
CK: Cytokeratin
CT: Computer Tomography
HE4: Human Epididymis Protein 4
HNF1β: Hepatocyte nuclear factor 1β
IL-1: Interleukin-1
P16: Multiple tumour suppressor 1
P53: Cellular tumour antigen
PET/CT: Positron Emission Tomography/Computed Tomography
PTEN: Phosphatase and tensin homologue deleted on chromosome 10
SCCA: Squamous cell carcinoma-associated antigen
